# Ensembling a Learned Volterra Polynomial with a Neural Network for Joint Nonlinear Distortions and Mismatch Errors Calibration of Time-Interleaved Pipelined ADCs

**DOI:** 10.3390/s25134059

**Published:** 2025-06-29

**Authors:** Yan Liu, Mingyu Hao, Hui Xu, Xiang Gao, Haiyong Zheng

**Affiliations:** College of Electronic Engineering, Ocean University of China, Qingdao 266404, China; liuyan5000@ouc.edu.cn (Y.L.); haomingyu@stu.ouc.edu.cn (M.H.); xh8187@stu.ouc.edu.cn (H.X.); gaoxiang1906@stu.ouc.edu.cn (X.G.)

**Keywords:** TI-pipelined ADC, calibration, nonlinear distortions, mismatch errors, Volterra polynomial, neural network

## Abstract

The inherent non-ideal characteristics of circuit components and inter-channel mismatch errors induce nonlinear amplitude and phase distortions in time-interleaved pipelined analog-to-digital converters (TI-pipelined ADCs), significantly degrading system performance. Limited by prior modeling, conventional digital calibration methods only correct partial errors, while machine learning (ML) approaches achieve comprehensive calibration at a high computational cost. This work proposes an ensemble calibration framework that combines polynomial modeling and ML techniques. The ensemble calibration framework employs a two-stage correction: a learned Volterra front-end performs forward mapping to compensate static baseline nonlinear distortions, while a lightweight neural network back-end implements inverse mapping to correct dynamic nonlinear distortions and inter-channel mismatch errors adaptively. Experiments conducted on TI-pipelined ADCs show improvements in both the spurious-free dynamic range (SFDR) and signal-to-noise and distortion ratio (SNDR). It is noteworthy that in two ADCs fabricated using 40 nm CMOS technology, the 12-bit, 3000 MS/s silicon-validated four-channel TI-pipelined ADC exhibits SFDR and SNDR improvements from 35.47 dB and 35.35 dB to 79.70 dB and 55.63 dB, respectively, while the 16-bit, 1000 MS/s silicon-validated four-channel TI-pipelined ADC demonstrates an enhancement from 38.62 dB and 40.21 dB to 80.90 dB and 62.43 dB, respectively. Furthermore, a comparison with related studies reveals that our method achieves comprehensive calibration performance for wide-band inputs while substantially reducing computational complexity, requiring only 4.4 K parameters and 8.57 M floating-point operations per second (FLOPs).

## 1. Introduction

The rapid development of radio frequency sensing devices and measurement systems has driven an increasing demand for high-performance ADCs. In particular, TI-pipelined ADCs, due to their high resolution and high-speed sampling capabilities, have become an ideal choice for applications requiring precise and rapid signal conversion. However, the performance of TI-pipelined ADCs is constrained by several non-ideal factors. Among these, channel mismatches introduce persistent spurious signals, significantly degrading the dynamic performance of TI-pipelined ADCs. Moreover, non-ideal characteristics of circuit components, including amplifier nonlinearities, comparator transient errors, and charge leakage in sampling circuits, lead to cumulative nonlinear distortions during signal conversion. Furthermore, physical defects in semiconductor devices, such as capacitance mismatches, further constrain the precision of signal sampling. Despite advancements in analog optimization techniques, significant challenges remain in mitigating the nonlinear effects caused by CMOS technology scaling and low supply voltage scenarios.

In response to these challenges, digital calibration technologies have been extensively developed in recent years due to their adaptability to process and voltage variations. For instance, ordered element matching (OEM) calibration is an improved method for dynamic element matching (DEM) technology [1,2], mainly used for compensating unit errors in analog-to-digital converters (ADCs) to improve linearity and performance; moreover, it can accurately reduce mismatch problems caused by manufacturing process deviations. Also, statistical and signal correlation-based methods [3,4,5,6] are widely employed to estimate nonlinear distortions and mismatch errors. When combined with least mean squares (LMS) algorithms, these methods enable ADC calibration in the digital domain [7]. Additionally, pseudo-random signals with well-defined characteristics can be injected into the ADC input to identify and estimate nonlinear distortions and mismatch errors [8,9,10], which are then corrected using adaptive algorithms. However, constrained by their reliance on statistical independence assumptions and linear time-invariant models, these methods achieve only partial error calibration. Although polynomial modeling methods, such as Volterra polynomials, can model multi-order nonlinear distortions with memory effect in TI-pipelined ADCs through generalized nonlinear expressions, their fixed coefficients fail to track frequency-dependent dynamic nonlinear distortions [11]. Furthermore, LMS-based adaptive coefficient updates require ideal reference signals, typically necessitating additional high-precision reference ADCs, which are often impractical to implement in applications.

Recently, ML techniques have inspired a prior-free approach for ADC calibration. By leveraging diverse neural network architectures, ML-based methods autonomously realize the nonlinear mapping between ADC output and ideal ground-truth values through backpropagation training, eliminating the need for explicit error modeling or prior assumptions. Specifically, fully connected neural networks (FCNNs) excel in scenarios with global data patterns and minimal spatial dependencies, as the architecture can theoretically capture overarching relationships across input features. This makes FCNNs a popular choice for correcting diverse errors, including nonlinear distortions and mismatch errors [12,13,14,15,16,17]. Conversely, when dealing with errors possessing spatial or temporal correlation structures, convolutional neural networks (CNNs) show superior performance, as evidenced by their applications for correcting timing skew errors in TIADCs [18,19]. However, due to their increased computational complexity, both FCNNs and CNNs face significant challenges when applied to ADC calibration for wide-band inputs. For example, Peng et al. proposed an FCNN-based calibration method, enabling joint calibration of nonlinear distortions and mismatch errors of a 12-bit 600 MS/s four-channel TI-pipelined ADC at 13.19 K parameters and 106.95 M FLOPs of computational complexity [17]. Additionally, Lu et al. trained a CNN-based nonlinearity calibration network (NCN) and an FCNN-based mismatch calibration network (MCN) to calibrate nonlinear distortions and mismatch errors of a 12-bit 5400 MS/s four-channel TIADCs at 51.4 K parameters and 103.55 M FLOPs of computational complexity [19]. The significant computational overhead of ML-based calibration methods presents a key implementation barrier for resource-constrained devices, demanding careful co-optimization of error correction accuracy and processing efficiency.

Actually, ML-based methods demonstrate strong capability for modeling complex nonlinear errors, including high-order intermodulation and time-varying nonlinear distortions through universal approximation. However, they require exponentially growing parameters and computational resources as error dimensionality increases. By comparison, Volterra polynomial-based methods offer precise characterization of main-path nonlinear distortions, such as amplifier nonlinear characteristics, through compact parametric representations, with physically meaningful kernel functions. This inherent complementarity inspires our ensemble modeling strategy: initial deterministic nonlinear components are modeled by polynomial basis functions, followed by a lightweight neural network processing residual dynamic errors. The approach delivers enhanced calibration accuracy alongside substantially improved computational efficiency. Experimental results demonstrate that our ensemble framework achieves efficient TI-pipelined ADC calibration, delivering comparable performance to existing neural network-based approaches while significantly reducing computational complexity.

The rest of this paper is organized as follows: Section 2 analyzes error mechanisms of TI-pipelined ADCs. Section 3 presents our ensemble calibration theory. Section 4 details the dataset and the training implementation. Section 5 evaluates experimental performance and hardware implementation. Finally, Section 6 concludes this work.

## 2. Comprehensive Analysis of Nonlinear Distortions and Mismatch Errors in TI-Pipelined ADCs

This section analyzes two critical types of errors in TI-pipelined ADCs, nonlinear distortions and mismatch errors. Although they share similar circuit-level behavioral characteristics, these errors demonstrate fundamentally different manifestations and system-level impacts. From a component-level perspective, we systematically investigate their origins and establish an analytical framework to guide calibration strategy development.

### 2.1. Nonlinear Distortions

Nonlinear distortions in TI-pipelined ADCs refer to signal fidelity degradation caused by non-ideal component behaviors during conversion, primarily comprising sampling nonlinearity and quantizer nonlinearities within the input sampling network. Regarding the sampling nonlinearity, we focus on the unavoidable sampling switches in most ADCs. For quantizer nonlinearities, we concentrate on the first stage, the multiplying digital-to-analog converter (MDAC) in pipelined ADCs, which significantly impacts the converter’s accuracy. Factors affecting the linearity of sampling switches include driver impedance, the tracking nonlinear distortion, and charge injection during sampling [20,21]. In ADCs, a reset switch is typically employed to manage capacitor charge states. Following the hold phase, this switch briefly resets capacitors prior to their connection to the input interface. Failure to reset these capacitors, coupled with insufficient settling time during the sampling phase, induces nonlinear charge injection that significantly degrades sampling linearity. At high sampling rates, the abbreviated sampling window generally prevents adequate stabilization of this nonlinear charge injection. The MDAC is the core functional module in pipelined ADCs, responsible for signal quantization, residual generation, and transfer. Its design directly influences the pipelined ADC’s linearity, noise performance, and conversion speed.

**Figure 1:**
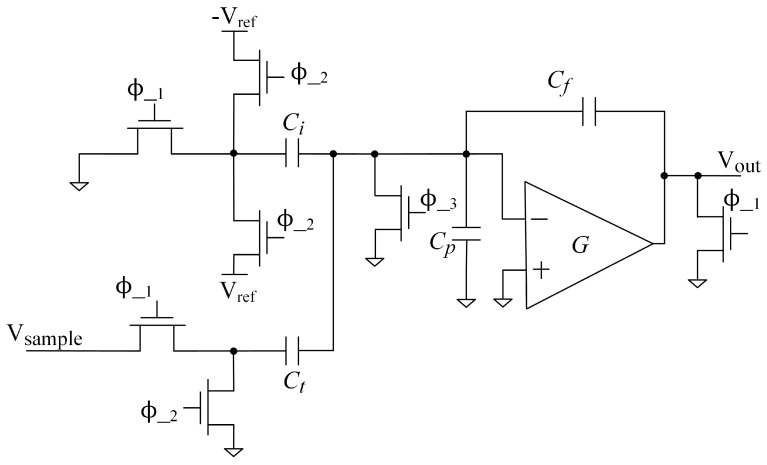
A split-capacitance MDAC.

A typical MDAC comprises a sampling network, a feedback DAC, and a residue amplifier. The sampling network and feedback DAC can either share the same capacitor array or utilize separate capacitor networks. Its operation can be decomposed into two stages: sampling and residual amplification. In an ideal scenario, the output of an MDAC with a split-capacitance network can be expressed as follows:

(1) $$\begin{array}{c} V_{\mathrm{out}}\left[n\right]=G\left(V_{\mathrm{sample}}\left[n\right]-V_{\mathrm{DAC}}\left[n\right]\right), \end{array}$$

where $V_{\mathrm{sample}}\left[n\right]$ is the ideal output of the sampling switch at the current time, $V_{\mathrm{out}}\left[n\right]$ is the output of the MDAC, $V_{\mathrm{DAC}}\left[n\right]$ is the quantized voltage value of the current stage, and *G* is the ideal gain of the residual amplifier. Due to factors such as manufacturing processes and charge release during switch transitions, sampling capacitor mismatch and charge injection errors introduced by switches can be introduced during the sampling and DAC conversion process, ultimately leading to changes in the capacitance values of the DAC capacitor array and the sampling capacitor array, further exacerbating the nonlinearity of the circuit. In this discussion, we analyze the distortion effects in a split-capacitance-based MDAC. As illustrated in Figure 1, when switch transistors α activate, a conduction channel forms at the gate oxide–channel interface. Charge accumulates within the MOS inversion layer during conduction. Upon switch deactivation, this channel charge partitions between source and drain terminals, subsequently injecting into downstream capacitors. Variations in injected charge modify capacitor node voltages, inducing capacitor mismatch errors within the array that exacerbate MDAC nonlinear distortion. When considering the mismatch scenario with split-capacitance networks, Equation (1) can be re-expressed as follows:

(2) $$\begin{array}{c} V_{\mathrm{out}}\left[n\right]=G\left(\frac{C_{t}+\Delta C_{t}}{C_{f}+\Delta C_{f}}V_{\mathrm{sample}}\left[n\right]-\frac{\sum_{i=1}^{m}D_{i}V_{refi}\left(C_{i}+\Delta C_{i}\right)}{C_{f}+\Delta C_{f}}\right), \end{array}$$

where $C_{i}$ represents the DAC capacitor array, $C_{t}$ denotes the sampling capacitor, $C_{f}$ is the feedback capacitor, and $C_{p}$ is the parasitic capacitance. ΔC represents the capacitance value offset caused by sampling capacitor mismatch and charge injection error related to codewords, ultimately resulting in severe DAC static nonlinear error. However, the finite gain and bandwidth of the residue amplifier, as well as its nonlinear characteristics, introduce nonlinear gain distortion into the overall output. The voltage transfer function can be derived as follows [15]:

(3) $$\begin{array}{c} V_{\mathrm{out}}\left[n\right]=\frac{A\beta }{1+A\beta }\left(\frac{C_{t}+\Delta C_{t}}{C_{f}+\Delta C_{f}}V_{\mathrm{sample}}\left[n\right]-\frac{\sum_{i=1}^{m}D_{i}V_{refi}\left(C_{i}+\Delta C_{i}\right)}{C_{f}+\Delta C_{f}}\right)\left(1-e^{-\frac{t}{\tau }}\right)\\ A=A_{0}+A_{1}V_{\mathrm{sample}}\left[n\right]+A_{2}V_{\mathrm{sample}}^{2}\left[n\right]+A_{3}V_{\mathrm{sample}}^{3}\left[n\right]+O\left(V_{\mathrm{sample}}^{3}\left[n\right]\right), \end{array}$$

where *A* is the open-loop gain coefficient of the residue amplifier, β is the feedback coefficient, *t* is the amplifier establishment time, and τ is the small-signal time constant. However, the finite gain of the operational amplifier introduces residual charges on the capacitors connected to the virtual ground, leading to unintended coupling between channels via memory effects [20,22]. Consequently, the historical state of preceding signals influences the current sampling cycle. When accounting for these memory effects and noise-induced distortion, the voltage transfer function can be expressed as follows:

(4) $$\begin{array}{cc} V_{\mathrm{out}}\left[n\right]= & \frac{A\beta }{1+A\beta }(\frac{C_{t}+\Delta C_{t}}{C_{f}+\Delta C_{f}}V_{\mathrm{sample}}\left[n\right]-\frac{\sum_{i=1}^{m}D_{i}V_{refi}\left(C_{i}+\Delta C_{i}\right)}{C_{f}+\Delta C_{f}}\\ & +\frac{\alpha C_{p}V_{\mathrm{out}}\left[n-1\right]}{C_{f}+\Delta C_{f}})\left(1-e^{-\frac{t}{\tau }}\right)+V_{n}, \end{array}$$

where $V_{n}$ is the output noise voltage and α is the memory effect coefficient induced by parasitic capacitance. Given that $V_{\mathrm{out}}\left[n\right]$ is determined by $V_{\mathrm{sample}}\left[n\right]$ and $V_{\mathrm{out}}\left[n-1\right]$, and that $V_{\mathrm{out}}\left[n-1\right]$ is, in turn, determined by $V_{\mathrm{sample}}\left[n-1\right]$ and $V_{\mathrm{out}}\left[n-2\right]$, the output voltage at the current time can be approximately expressed as follows:

(5) $$\begin{array}{cc} V_{\mathrm{out}}\left[n\right]\approx & G_{10}+G_{11}V_{\mathrm{sample}}\left[n\right]+G_{12}V_{\mathrm{out}}\left[n-1\right]+G_{13}V_{\mathrm{sample}}^{2}\left[n\right]+G_{14}V_{\mathrm{sample}}\left[n\right]V_{\mathrm{out}}\left[n-1\right]\\ & +G_{15}V_{\mathrm{sample}}^{3}\left[n\right]+G_{16}V_{\mathrm{sample}}^{2}\left[n\right]V_{\mathrm{out}}\left[n-1\right]+V_{n}, \end{array}$$

(6) $$\begin{array}{cc} V_{\mathrm{out}}\left[n-1\right]\approx & G_{20}+G_{21}V_{\mathrm{sample}}\left[n-1\right]+G_{22}V_{\mathrm{out}}\left[n-2\right]+G_{23}V_{\mathrm{sample}}^{2}\left[n-1\right]+\ldots , \end{array}$$

where the influence of $V_{\mathrm{out}}\left[n-2\right]$ on $V_{\mathrm{out}}\left[n\right]$ can be neglected due to the coupling effects among the coefficients; the output voltage at the current time can be approximately expressed as follows:

(7) $$\begin{array}{cc} V_{\mathrm{out}}\left[n\right]\approx & H_{0}+H_{1}V_{\mathrm{sample}}\left[n\right]+H_{2}V_{\mathrm{sample}}\left[n-1\right]+H_{3}V_{\mathrm{sample}}^{2}\left[n\right]\\ \; & +H_{4}V_{\mathrm{sample}}\left[n\right]V_{\mathrm{sample}}\left[n-1\right]+H_{5}V_{\mathrm{sample}}^{2}\left[n-1\right]+H_{6}V_{\mathrm{sample}}^{3}\left[n\right]\\ \; & +H_{7}V_{\mathrm{sample}}^{2}\left[n\right]V_{\mathrm{sample}}\left[n-1\right]+H_{8}V_{\mathrm{sample}}\left[n\right]V_{\mathrm{sample}}^{2}\left[n-1\right]+H_{9}V_{\mathrm{out}}^{3}\left[n-1\right]+V_{n}. \end{array}$$

**Figure 2:**
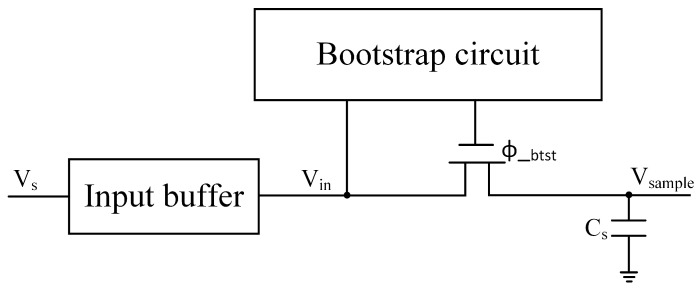
A sampling network with a bootstrapped input switch and input buffer.

The sampling stage at the front-end of high-speed ADCs, typically constructed from switch–capacitor circuits, is usually the limiting factor for their high-frequency linearity performance. Due to the finite speed of the sampling switches, frequency-dependent nonlinear errors are introduced, reducing the ADC’s performance at high input frequencies. As shown in Figure 2, the primary sources of error in this stage are input-dependent charge injection and track-and-hold nonlinearities caused by the impedance modulation of the sampling switch, which introduce frequency-dependent dynamic nonlinearities [23,24,25]. Since the charge on the capacitor at the end of the sampling phase is converted into a discrete-time sample, the relationship between the discrete-time samples before and after sampling can be expressed as follows [23]:

(8) $$\begin{array}{c} V_{\mathrm{in}}\left[n\right]=V_{\mathrm{sample}}\left[n\right]+\left(A_{4}+A_{5}V_{\mathrm{sample}}\left[n\right]+A_{6}V_{\mathrm{sample}}^{2}\left[n\right]+\ldots \right)\frac{dV_{\mathrm{sample}}\left[n\right]}{dt}. \end{array}$$

### 2.2. Mismatch Errors

Variations in the electronic structures of sub-ADCs lead to offset, gain, nonlinear, and timing skew mismatches in TI-pipelined ADCs. The mismatches lead to interleaving spurs, which worsen with increasing input frequency. Nonlinear mismatch arises from differing nonlinear effects across channels. Similar to the other three types of mismatch distortion, this nonlinear discrepancy causes channel mismatch distortion in the TI-pipelined ADCs. Once the nonlinearities of individual channels are calibrated, this nonlinear mismatch can be effectively eliminated. Therefore, here we focus on discussing the other three types of mismatch errors.

#### 2.2.1. Offset Mismatch and Gain Mismatch

Calibration for offset and gain mismatches is relatively straightforward, typically involving the subtraction of voltage offsets and gain adjustment in sub-channels. When there are gain mismatch and offset mismatch errors in the circuit, there exists a gain term and an offset term between the input and output of the *m*-th ADC, which can be expressed as follows:

(9) $$\begin{array}{c} V_{\mathrm{out}}\left[n\right]=\left(1+\Delta G\right)V_{\mathrm{in}}\left[n\right]+V_{o}, \end{array}$$

where ΔG is the gain coefficient introduced by the gain of the sub-ADC, and $V_{o}$ is the offset coefficient between the output signal and the input signal.

#### 2.2.2. Timing Skew Mismatch

Sampling time skew refers to the phenomenon where multi-channel signals should theoretically be sampled simultaneously at the same time; however, due to hardware, circuit, or clock allocation reasons, there is a small time difference (known as skew) between the sampling times of each channel, which means that the sampling times are not completely synchronized. When the timing skew exists in the sampling switch, the relationship between the input and output can be expressed as follows:

(10) $$\begin{array}{c} V_{\mathrm{in}}\left[n\right]=V_{\mathrm{sample}}\left[n\right]+\Delta t\frac{dV_{\mathrm{sample}}\left[n\right]}{dt}. \end{array}$$

In the previous sub-section, it is known that the output of the switch circuit contains frequency-dependent dynamic nonlinear errors, as expressed by Equation (8). This frequency dependence can be represented using the signal derivative. Meanwhile, the sampling time skew errors of the sampling switch, which is related to time and the signal derivative, can be expressed as Equation (10). This error related to signal derivatives is parallel to dynamic nonlinear distortion. By combining these two dynamic errors, we can obtain the representation of the dynamic error of the switch circuit in Equation (11) and thus derive our subsequent separation calibration architecture for TI-pipelined ADC errors. The charge on the capacitor at the end of the tracking phase of the sampling switch circuit is converted into a discrete-time sample. Considering both the dynamic nonlinear distortion and the sampling time skew, the relationship between the pre-sampling and post-sampling discrete-time samples can be expressed as follows:

(11) $$\begin{array}{c} V_{\mathrm{in}}\left[n\right]=V_{\mathrm{sample}}\left[n\right]+\left(\Delta t+A_{4}+A_{5}V_{\mathrm{sample}}\left[n\right]+A_{6}V_{\mathrm{sample}}^{2}\left[n\right]+\ldots \right)\frac{dV_{\mathrm{sample}}\left[n\right]}{dt}. \end{array}$$

### 2.3. Comprehensive Analysis

After the above analysis, when an external signal source is fed into multiple channels, clock signal delays, nonlinear effects of the switch circuit, and quantization circuits lead to the fusion of nonlinear distortions and channel mismatch errors in the TI-pipelined ADC’s output. The switch circuit and similar structures are influenced by the analog circuitry’s frequency response characteristics. Input signals with different frequencies manifest varying degrees of nonlinear distortions and mismatch errors in the TI-pipelined ADC’s output. For single-channel nonlinear distortion, second- and third-order harmonics dominate.

**Figure 3:**
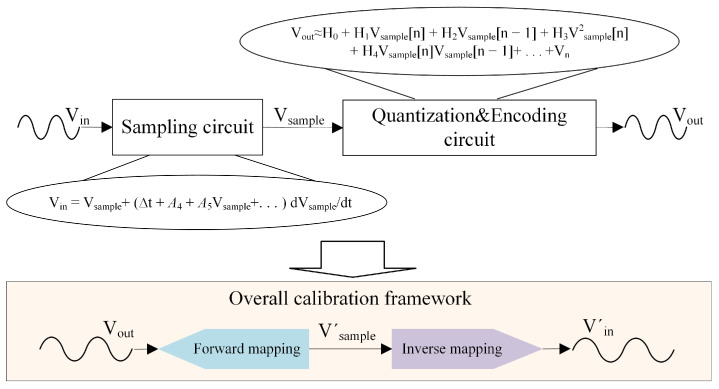
Inspiration diagram for TI-pipelined ADC calibration algorithm.

In Equation (7), the quantizer’s output is related to its input (the output of the sampling circuit) through a functional relationship, representing a forward mapping. Conversely, in Equation (11), the sampling circuit’s input and output are related via an inverse mapping, where the output signal represents the input signal. This reciprocal relationship inspires us, as illustrated in Figure 3, with regard to a calibration model being able to be constructed by exploiting these different mappings and enabling effective correction of distortions. Because the response of the electronic components in the quantization circuit exhibits minimal variation under different input signals, the coefficients in Equation (7) vary only slightly. In contrast, the switch circuit associated with Equation (11) is significantly more affected. Consequently, nonlinear distortions can be distinctly classified into static baseline nonlinear distortion and dynamic nonlinear incremental distortion, corresponding, respectively, to the two inverse mapping processes described above.

Based on this, we propose an ensemble model for TI-pipelined ADC calibration that combines forward and inverse mapping processes, as shown in Figure 3. First, by employing the adaptive moment estimation (Adam) optimization algorithm, which is based on the gradient descent method, to learn explicit polynomial equations, our method calibrates the static baseline distortion of each sub-ADC based on forward mapping processes. This process effectively preserves the dynamic nonlinear incremental distortion and dynamic channel mismatch errors within the data. Second, we combine derivative information with sequential data since integrating feature and derivative information can efficiently capture subtle variations, reveal underlying patterns or anomalies, and reflect fluctuation trends. Utilizing the gradient information of the loss function, we minimize the discrepancy between ground-truth and actual values based on an inverse mapping process, thereby calibrating the dynamic nonlinear incremental distortions and dynamic channel mismatch errors.

## 3. Learned Volterra–Neural Network Ensemble Model

### 3.1. Overall Architecture

**Figure 4:**
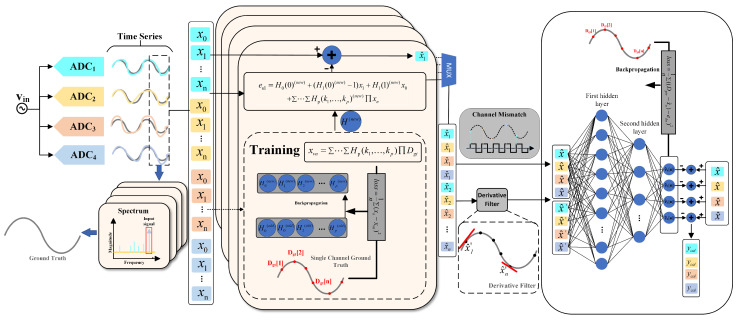
Overall architecture of the ensemble model and the proposed training process.

This paper proposes an ensemble model to address the nonlinear distortions and mismatch errors in TI-pipelined ADCs, as shown in Figure 4. The model employs a two-stage processing mechanism: the Volterra front-end first eliminates static baseline distortions via polynomial modeling with Adam-optimized kernel coefficients, inherently leaving uncorrected dynamic nonlinear distortions and inter-channel mismatches in its residuals. These residuals are then passed to the lightweight FCNN back-end. The Volterra front-end and FCNN back-end achieve complementary functionality: Volterra reduces static nonlinear distortions with low computational overhead, while the FCNN corrects residuals through learned cross-channel correlations. Details of the two-stage processes are presented in the following subsections.

### 3.2. Learned Volterra Front-End: Static Baseline Distortion Forward Mapping

The Volterra series is a robust mathematical framework for modeling and analyzing nonlinear systems, enabling the characterization of behaviors beyond the limitations of linear and memoryless models. Notably, it excels in capturing higher-order harmonic nonlinearities with memory effects, establishing its significance in nonlinear system studies. The continuous-time-domain representation of the Volterra series is given as follows:

(12) $$\begin{array}{cc} Y(t) & =\sum_{p=0}^{P}\int_{-\infty }^{\infty }\ldots \int_{-\infty }^{\infty }H_{p}\left(\tau_{1},\ldots ,\tau_{n}\right)\prod_{i=1}^{p}X\left(t-\tau_{i}\right)d\tau_{i}, \end{array}$$

where X(t) and Y(t) denote the system’s input and output at time *t*, respectively, *p* is the Volterra series order, and $H_{p}$ represents the *p*-th order continuous-time Volterra kernel. For discrete-time systems, the formulation becomes:

(13) $$\begin{array}{cc} Y[n] & =\sum_{p=0}^{P}\sum_{k_{1}=-\infty }^{\infty }\ldots \sum_{k_{p}=-\infty }^{\infty }H_{p}\left[k_{1},\ldots ,k_{p}\right]\prod_{i=1}^{p}X\left[n-k_{i}\right]. \end{array}$$

From Equation (7) and Equation (13), we observe that the Volterra polynomial can capture the memory effect nonlinear distortions introduced by the MDAC.

**Figure 5:**
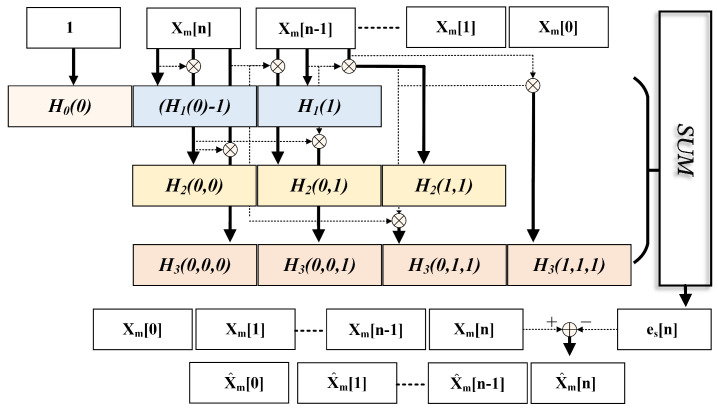
Block diagram of truncated third-order Volterra implementation for channel-m ADC calibration.

Given that MDAC exhibits weak nonlinearity dominated by low-order harmonics, the previous experimental results in Table 1 indicate that Volterra cannot effectively calibrate second-order and third-order nonlinear distortions when the order is below the third order and the storage depth is below the second order. Increasing the storage depth or order afterwards does not significantly improve ADC performance, and the static nonlinear fundamental distortion has been fully calibrated. In addition, when increased to the fourth order, about 10 DSPs will be added, and the four channels will further increase resource consumption. Therefore, we adopt a truncated Volterra polynomial with third-order nonlinearity and a memory depth of two, yielding the following simplified representation:

(14) $$\begin{array}{cc} y[n]= & \sum_{p=0}^{3}\sum_{k_{1}=0}^{1}\ldots \sum_{k_{p}=k_{p-1}}^{1}H_{p}\left[k_{1},\ldots ,k_{p}\right]\prod_{i=1}^{p}x\left[n-k_{i}\right]\\ = & H_{0}+H_{1}\left\{x\left[n\right],x\left[n-1\right]\right\}+H_{2}{\left\{x\left[n\right],x\left[n-1\right]\right\}}^{2}\\ \; & +H_{3}{\left\{x\left[n\right],x\left[n-1\right]\right\}}^{3}, \end{array}$$

where $H_{p}$ (p∈{0,1,2,3}) denotes the *p*-th order Volterra kernel coefficients, x[n] represents the ideal sampled value at time instant *n*, and x[n−1] corresponds to the previous time sample. Given that the nonlinearity of MDAC and superimposed dynamic errors exhibit frequency-dependent variations, which would otherwise require coefficient adaptation, we employ backpropagation to train a set of globally optimal Volterra coefficients to extract static baseline distortion of each sub-ADC, thereby eliminating the need for adaptive updates. As shown in Figure 5, the *m*-th ADC’s static errors $e_{s}\left[n\right]$ can be expressed as follows:

(15) $$\begin{array}{cc} e_{s}\left[n\right]= & H_{0}\left(0\right)+\left[H_{1}\left(0\right)-1\right]x_{m}\left[n\right],H_{1}\left(1\right)x_{m}\left[n-1\right]\\ \; & +H_{2}{\left\{x_{m}\left[n\right],x_{m}\left[n-1\right]\right\}}^{2}+H_{3}{\left\{x_{m}\left[n\right],x_{m}\left[n-1\right]\right\}}^{3}. \end{array}$$

We employ a third-order Volterra model with a memory depth of 2, each of which corresponds to a sub-channel of the TI-pipelined ADC. In total, only 40 parameters are required for all sub-channels. Within the PyTorch 2.5.1 framework, we construct the corresponding Volterra polynomials and use the Adam optimizer to train and update the optimal Volterra kernel values through backpropagation. These kernel values describe the static nonlinear behavior of the full-bandwidth sub-ADC system and are used for the pre-calibration of static nonlinear distortion. The ground truth is obtained using the method described in Section 4. As shown in Figure 4, by using the values of two consecutive samples as inputs, the ideal calibration values for subsequent samples can be determined. The pipelined architecture enables the real-time output of pre-calibrated signals.

### 3.3. Neural Network Back-End: Dynamic Nonlinear Distortion and Mismatch Error Inverse Mapping

Neural networks excel at nonlinear representation and complex mapping. Theoretically capable of approximating any nonlinear function, neural networks can fully characterize ADC nonlinear systems when trained with appropriate data and models. Moreover, the calibration process can be expressed with the following equation:

(16) $$\begin{array}{cc} y_{\mathrm{cal}}\left[n\right] & =\hat{x}\left[n\right]-F_{NN}\left(\hat{x}\left[n\right]\right)\\ \; & =\hat{x}\left[n\right]-{\hat{e}}_{m}\left[n\right]\\ \; & =\hat{x}\left[n\right]-\left(x\left[n\right]\Delta_{Gd}+\alpha_{1d}x_{\mathrm{in}}^{2}+\alpha_{2d}x_{\mathrm{in}}^{3}+V_{mis}\right), \end{array}$$

where $y_{cal}\left[n\right]$ is the ground truth of the ADC at the current time, $F_{NN}\left(\right)$ represents the inverse mapping of the ADC system by the neural network, ${\hat{e}}_{m}\left[n\right]$ represents the dynamic nonlinear distortion and mismatch errors of the sampled data $x_{m}\left[n\right]$ in the *m*-th channel, and $\hat{x}\left[n\right]$ is the calibartion data by the Volterra model. We do not directly use neural networks to map the ADC system. We utilize neural networks to map the dynamic errors of the ADC, enabling effective calibration.

**Figure 6:**
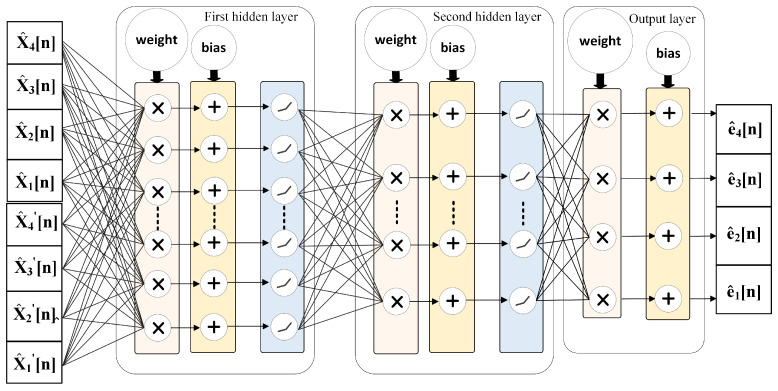
Block diagram of proposed neural network model.

As shown in Figure 6, we design an FCNN to model and calibrate the dynamic nonlinear and mismatch errors in a TI-pipelined ADC, primarily stemming from intra-channel nonlinearities and inter-channel timing skews. To improve sequence deviation modeling while reducing network complexity, we augment the input data with its derivatives as local features. By leveraging gradient-based optimization, we minimize the discrepancy between predicted and actual values. Thus, the FCNN serves as an error-fitting module, effectively capturing the deviations between raw and ideal ADC output.

The FCNN model consists of an input layer, two hidden layers, and an output layer. In the hidden layers, each neuron is connected to all neurons in the preceding and following layers. The input layer contains 8 nodes, of which the first 4 neurons represent the original pre-calibrated data points, and the latter 4 neurons contain the corresponding first-order derivative information; the hidden layers have 256 and 8 neurons, respectively, while the output layer includes 4 neurons, representing the differences between pre-calibrated data and ideal data. We employ the Leaky ReLU nonlinear activation function throughout the network to introduce the ability to learn nonlinearity. Finally, error compensation is achieved through residual connections, completing the entire calibration process. Compared to traditional ML methods, this architecture significantly reduces the computational complexity required for ADC calibration.

## 4. Dataset Construction and Training of the Ensemble Model

A key step in the calibration process is analyzing the output sequence y[n] to construct a ground truth that ensures precise alignment with the input x[n]. While fast Fourier transform (FFT) methods are widely used in frequency-domain analysis, they often encounter significant spectrum leakage in practical applications. This phenomenon leads to inaccuracies in the spectral components, thereby preventing them from accurately reflecting the signal’s characteristics. Moreover, it affects the feature extraction process and may result in deviations in the model parameters. Therefore, we employ the time-shift phase spectrum calibration method [26]. This method enables the precise extraction of the signal’s phase, frequency, and amplitude information. However, extracting only single-tone signals is not enough. Therefore, when extracting the frequency-domain features of the effective components of multi-tone signals, we can define the frequency range of each signal spectral line. Taking a dual-tone signal as an example, this means that we can divide it into two small frequency intervals, each interval containing a spectral line. Therefore, we can measure their respective frequencies, phases, and amplitudes; retain the effective components when constructing the ground truth; and retain the corresponding effective components in the final mapping process. Upon completing the extraction process, we implement a sinusoidal signal fitting technique to construct Volterra’s ground truth x[n] and the neural network’s ground truth $y_{cal}\left[n\right]$. This approach effectively provides a high-quality data foundation for both neural network training and parameter estimation in polynomial models, and it enhances the reliability of model training.

Our proposed model is designed for error separation calibration of the TI-pipelined ADC. If we consider the first sub-ADC as the reference channel and construct the ground truth through an interleaved four-channel configuration, the data collected by the other channels will exhibit mismatch deviations relative to this ideal sequence. Such error aliasing can reduce the effectiveness of nonlinear calibration. To mitigate the impact of channel mismatch errors during the calibration of static baseline nonlinear distortions, we do not explicitly consider mismatch errors. Instead, we construct the ground truth for each individual channel independently, which effectively eliminates a portion of the second- and third-order nonlinear distortion and yields pre-calibrated data for each channel. These pre-calibrated data are interleaved in the second calibration stage to form the TI-pipelined ADC’s output data. This approach ensures that, within the ideal sequence, mismatch distortions are absent. By using this sequence as a benchmark, the influence of nonlinear distortions on mismatch calibration is significantly reduced, leading to more accurate correction of channel mismatch errors.

During the first stage of model training, the data collected from a four-channel TI-pipelined ADC were randomly sampled within the frequency range of [0,π], selecting 2800×0.7 frequency points. Each frequency contained 65,536 sampling points; thus, each channel consisted of 16,384 sampling points. The first 501 sampling points from each channel were extracted. When the memory depth was set to 2, a single frequency data point could be extended to form 500 training samples. Therefore, for a single-channel nonlinear calibration model, the total number of training samples is 2800×0.7×500. After the initial pre-calibration was completed, the pre-calibrated data from each channel were interleaved to reconstruct 2800 sets of pre-calibrated data, and derivatives were computed for each set, resulting in 2800 derivative datasets. In the second stage of training, these 2800 sets of pre-calibrated data, along with their corresponding derivative data, were randomly partitioned into training and testing sets at a ratio of 7:3. For each frequency point in the training set, 500 data samples were extracted. Due to the specific input format required by the neural network, every four features were sampled as a single input, resulting in 2000 features per frequency point for the 500 data samples.

The application methods of ground truth in these two stages differ, thus constituting two opposite model mapping processes. In the first stage of the learned Volterra-based calibration, the input to the model is the separated channel ground truth, with the output representing the single-channel acquisition data. After the model training in the first stage was completed, since the ground truth is not available directly from ADC chips, and the actual ground truth is required for calibration, we used the acquisition data as a surrogate for the ground truth to facilitate real-time pre-calibration at the ADC back-end. In the second stage of the FCNN-based calibration, the neural network model utilized the interleaved pre-calibrated data from multiple channels as input, with the output being the combined channel ground truth.

Both stages employed an initial learning rate of 0.01 during training, totaling 2000 epochs. A cosine annealing learning rate schedule was adopted to adjust the learning rate dynamically. The mean squared error (MSE) was used as the loss function during forward propagation, while the Adam optimizer facilitated parameter updates via backpropagation. The training continued until the model achieved satisfactory calibration performance.

## 5. Results and Discussion

To validate the effectiveness of the ensemble model illustrated in Figure 4, we conduct tests using a behavioral TI-pipelined ADC model built in Simulink and two silicon-validated ADCs. The silicon-validated ADCs include a 12-bit 3000 MS/s four-channel TI-pipelined ADC and a commercial 16-bit 1000 MS/s four-channel TI-pipelined ADC. To demonstrate the superiority of our ensemble model’s performance, we compare the calibration performance of these TI-pipelined ADCs before and after calibration, and we contrast these results with those reported in recent advanced work. Furthermore, to assess the advantages of our ensemble model regarding computational complexity, we select two authoritative and representative evaluation metrics: the number of model parameters and FLOPs [27,28,29]. These metrics help gauge model complexity by indicating the number of multiply–accumulate operations required to process an equal number of feature points during ADC calibration, standardized at 4096 points.

### 5.1. Simulation Results

In the simulation validation stage, we utilize a behavioral ADC model: a 12-bit 4000 MS/s four-channel TI-pipelined ADC model. To simulate a realistic calibration scenario for the TI-pipelined ADC, we incorporate various errors, including DAC capacitor errors, inter-stage redundancy amplifier gain errors, second-order and third-order nonlinear harmonic distortions, and channel mismatch distortions. Specifically, inter-stage gain variations are introduced, randomizing the amplifier gain within the range of $\left[0.99G_{ideal},1.01G_{ideal}\right]$, and DAC errors within the range of $\left[0.95DAC_{ideality},1.05DAC_{optical}\right]$.

**Figure 7:**
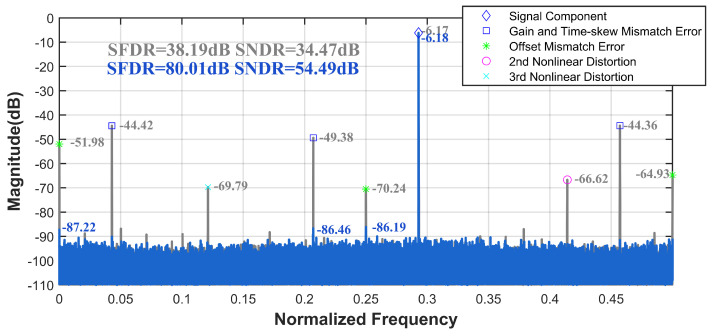
The spectrum before and after the ensemble model calibration with fin = 1.17 GHz of the 12-bit 4000 MS/s four-channel TI-pipelined ADC model.

**Figure 8:**
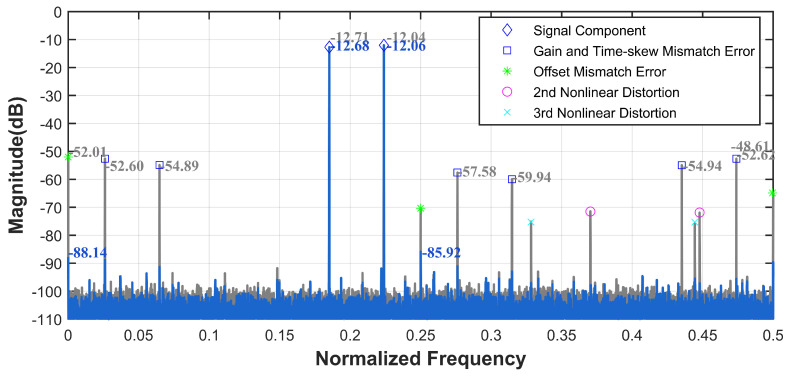
The spectrum before and after the ensemble model calibration with a two tone signal of the 12-bit 4000 MS/s four-channel TI-pipelined ADC model.

Mismatch errors can be categorized into three types, gain mismatch, offset mismatch, and timing skew mismatch, with sampling time skew being the most significant contributor to overall errors. We introduce the aforementioned errors into the pipelined ADC, using it as the sub-ADC for the TI-pipelined ADC. Each channel is subjected to sampling time skews within the range of $\left[0,-2.6\%T_{s},-2.2\%T_{s},1.1\%T_{s}\right]$. Utilizing our ground-truth acquisition method, we construct a calibration dataset with randomized phases within the range of [0,2π] at the first Nyquist sampling frequency. After training, we test the model with wide-band input signals. As shown in Figure 7, when the input signal frequency was set to 1.17 GHz, the SFDR and SNDR of the TI-pipelined ADC significantly improved from 38.19 dB and 34.47 dB to 80.01 dB and 54.49 dB, respectively. Additionally, we evaluate the model’s performance using randomly generated dual-tone signals. The spectral comparisons before and after calibration, as depicted in Figure 8, demonstrate that significant performance enhancements are achieved without altering the model structure, confirming the ensemble model’s efficacy in improving the calibration of multi-tone signals.

### 5.2. Measurement Results

This section presents the calibration of two silicon-verified TI-pipelined ADCs using the proposed ensemble model. The external circuit sampling time delays inherent in TI-pipelined ADCs result in imperfect matching of multi-channel sampling times, introducing timing skew mismatch errors into the output signal [30]. Consequently, these errors can lead to significant inaccuracies in the TI-pipelined ADC’s output, potentially exceeding the dynamic nonlinear errors of the sub-ADCs. In the output spectrum of the TI-pipelined ADCs, these inaccuracies primarily manifest at positions related to the input signal frequency and the sampling rate.

**Figure 9:**
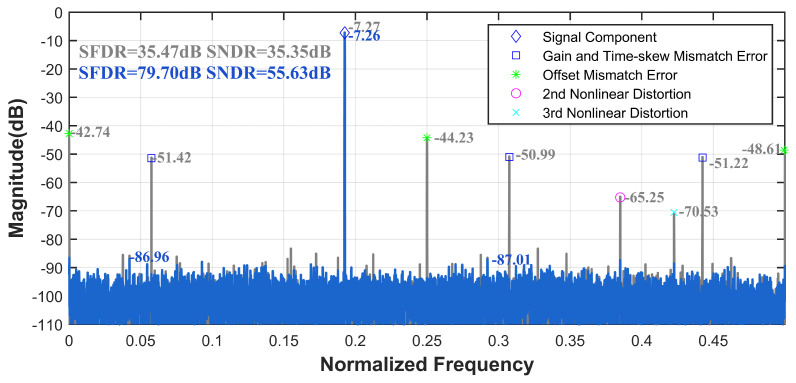
Measured output spectrum before and after the ensemble model calibration with fin = 577.5 MHz of the 12-bit 3000 MS/s four-channel TI-pipelined ADC.

For the 12-bit 3000 MS/s four-channel TI-pipelined ADC, Figure 9 illustrates the spectrum comparisons before and after calibration at input frequencies of 577.5 MHz. The result indicates a successful elimination of harmonics and spurious signals caused by nonlinear distortions in the single-channel and mismatch errors introduced by multi-channel interleaving. For the signal input of 577.5 MHz, the SFDR and SNDR improved from 35.47 dB and 35.35 dB to 79.70 dB and 55.63 dB, respectively. This demonstrates that the proposed ensemble model effectively calibrates the errors of the TI-pipelined ADC.

**Figure 10:**
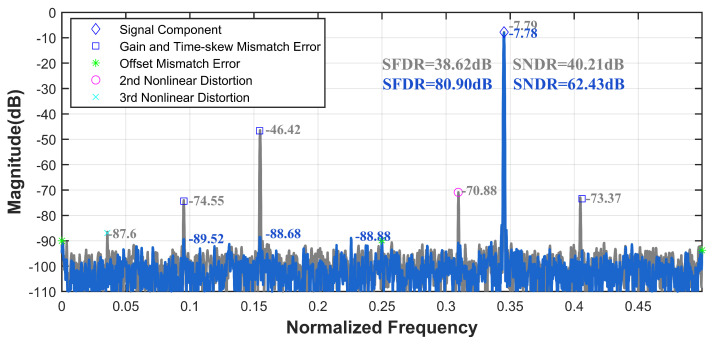
Measured output spectrum before and after the ensemble model calibration with fin = 345.2 MHz of the proposed 16-bit 1000 MS/s four-channel TI-pipelined ADC.

**Figure 11:**
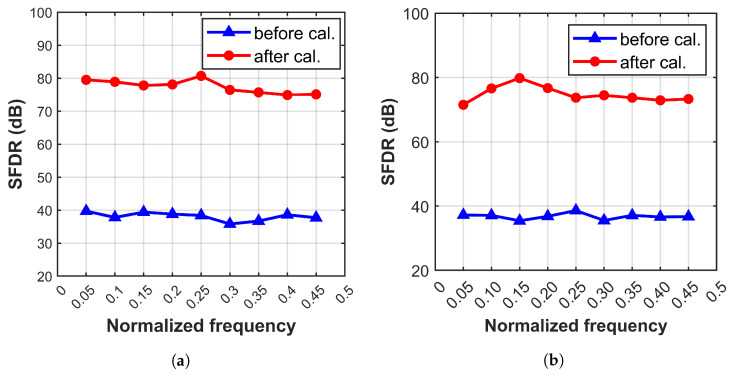
The calibration performance of the TI-pipelined ADCs with the input of wide-band signals. Panel (**a**) shows a 12-bit 4000 MS/s four-channel TI-pipelined ADC model. Panel (**b**) shows a 12-bit 3000 MS/s four-channel TI-pipelined ADC.

To further validate the applicability of the ensemble model across various scenarios, calibration verification is conducted using a commercial 16-bit 1000 MS/s four-channel TI-pipelined ADC. Without altering the model size and FLOPs, improvements in SFDR and SNDR are observed from 38.62 dB and 40.21 dB to 80.90 dB and 62.43 dB under the input of 345.2 MHz, as shown in Figure 10. As shown in Figure 11, the calibration performance of the 12-bit 4000 MS/s four-channel TI-pipelined ADC model and the 12-bit 3000 MS/s four-channel TI-pipelined ADCs in the wide-band is outstanding. As shown in Table 2, a comparison of the performance enhancements of ADCs with respect to the complexity parameters of the model confirms that the proposed ensemble model offers beneficial calibration effects for system errors in TI-pipelined ADCs while maintaining low complexity.

### 5.3. Ablation Analysis of Our Ensemble Model

To demonstrate the superiority of our ensemble model, we conducted a series of ablation experiments on the TI-pipelined ADCs, including the following: (1) Calibration of the ADC output considering only static nonlinear distortion. (2) Calibration of the ADC output after addressing static nonlinear distortion, dynamic nonlinear distortion, and channel mismatch errors. The comparison results are shown in Table 3.

**Figure 12:**
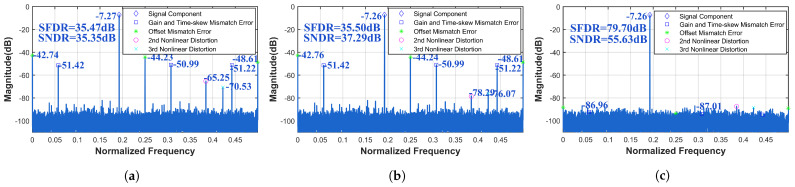
Measured output spectrum before and after calibration with fin = 577.5 MHz with the 3000 MS/s TI-pipelined ADC (**a**) before calibration, (**b**) after static nonlinear calibration, and (**c**) after calibration by the ensemble model.

**Figure 13:**
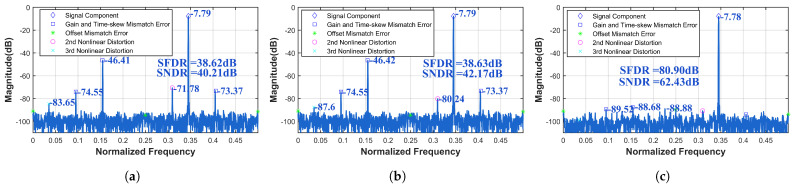
Measured output spectrum before and after calibration with fin = 344.2 MHz with the 1000 MS/s TI-pipelined ADC (**a**) before calibration, (**b**) after static nonlinear calibration, and (**c**) after calibration by the ensemble model.

Since mismatch errors have the greatest impact on the SFDR and SNDR, their differences are not readily apparent from the metrics themselves at this point. However, significant distinctions can be observed in Figure 12 and Figure 13. For the four-channel TI-pipelined ADC with a 12-bit resolution and 3000 MS/s sampling rate, calibrating only the static nonlinear distortion, the SFDR and SNDR increased from 35.47 dB and 35.35 dB to 35.50 dB and 37.29 dB, respectively. The calibration result indicates that the learned Volterra model performs poorly in wide-band signal calibration. After calibration of static nonlinear distortion, dynamic nonlinear distortion, and channel mismatch errors, the SFDR and SNDR improved from 35.50 dB and 37.29 dB to 79.70 dB and 55.63 dB. For the four-channel TI-pipelined ADC with a 16-bit resolution and 1000 MS/s sampling rate, calibrating only the static nonlinear distortion, the SFDR and SNDR increased from 38.62 dB and 40.21 dB to 38.63 dB and 42.17 dB. After full calibration, including static nonlinear distortion, dynamic nonlinear distortion, and channel mismatch errors, the SFDR and SNDR improved from 38.63 dB and 42.17 dB to 80.90 dB and 62.43 dB.

Therefore, our ensemble model achieves optimal calibration performance, demonstrating that this approach maintains high performance while exhibiting excellent calibration effects with low complexity.

### 5.4. Hardware Implementation

**Figure 14:**
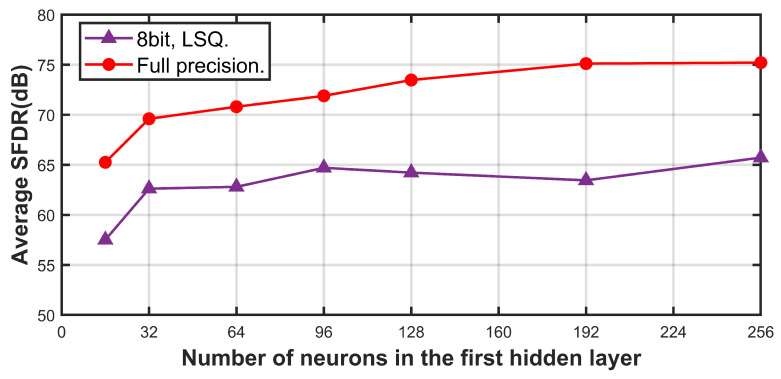
Calibration performance of the 1000 MS/s TI-pipelined ADC with different number of neurons under two types of data modalities.

We designed the proposed calibration structure using the Verilog hardware description language and implemented it on the Xilinx Zynq UltraScale+ MPSoC XCZU15EG to realize online calibration. In practical applications of the proposed calibration model, employing low-bit-width quantization methods and reducing the number of hidden layer neurons can significantly conserve computational hardware resources. Figure 14 illustrates the relationship between SFDR and the number of nodes in the first hidden layer for the commercial four-channel TI-pipelined ADC with a 16-bit resolution and 1000 MS/s sampling rate after calibration using a fixed set of neural network weights under various configurations of hidden layer neurons and quantization schemes. When using a full-precision scheme with 32 neurons, the average accuracy across the entire frequency band decreases by only 5 to 6 dB compared to a full-precision scheme with 256 neurons. After applying the learned step size quantization (LSQ) scheme [32], with both weights and inputs quantized to 8 bits, the SFDR can still reach 62 dB.

**Figure 15:**
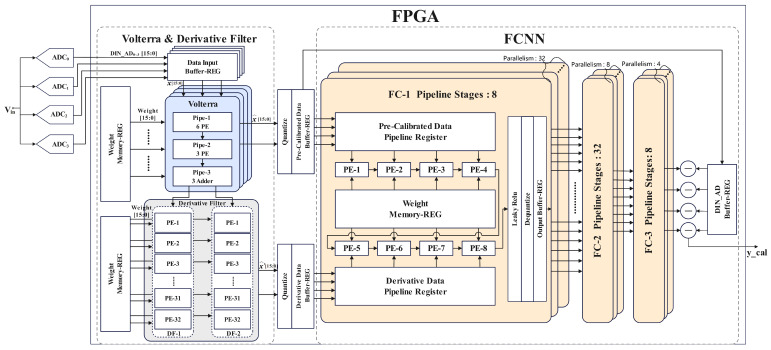
Block diagram of the ensemble model’s hardware implementation.

As depicted in Figure 15, the forward propagation calibration model was deployed on the FPGA platform. To synchronize with the 1000 MS/s sampling rate of the four-channel TI-pipelined ADC, a clock frequency of 250 MHz was utilized in the Volterra data processing unit, which is strictly aligned with the sampling rate of the single-channel pipelined ADC. In the derivative generation module, a clock frequency of 500 MHz was employed, achieving full pipelining by leveraging the properties of anti-symmetrical tap coefficients and zero intermediate coefficients. This optimization reduced the parallelism from 2 × 32 to 2 × 16. To support the implementation of high-frequency circuits, we adopted a multi-stage pipelined design for the multiply–accumulate (MAC) operations within each processing element (PE) unit. Specifically, the multiplication operations were structured with a five-stage pipeline, while the addition operations utilized a three-stage pipeline. Both multiplication and addition computations were executed using digital signal processors (DSPs), thereby eliminating the need for lookup table (LUT)-based adder implementations. This approach effectively reduced LUT resource consumption.

**Figure 16:**
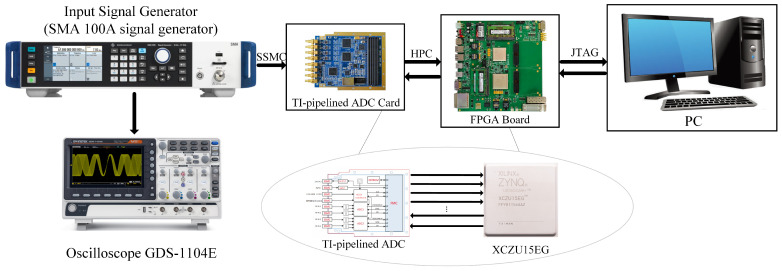
Hardware schematic diagram of TI-pipelined ADC acquisition system.

The Volterra module involves 20 multiplications and 9 additions. Due to the non-1:1 ratio of multiplications to additions, it was infeasible to fully implement all MAC operations via DSPs, as achieved in the neural network section. By applying factorization techniques, we compressed the original 20 multiplications to 12 and implemented 7 out of the 9 additions using DSPs, leaving only 2 additions to be handled by LUTs. Through these optimizations, we minimized the resource overhead of the Volterra module. Experimental results demonstrated that our design required only 3480 while achieving real-time calibration, a significant improvement over prior studies. The fully pipelined architecture further enhanced hardware resource utilization, enabling a lightweight circuit design. Figure 16 illustrates the overall ADC acquisition system setup and the electronic equipment used for the calibration circuitry. The total resource utilization is listed in Table 4. Moreover, the total on-chip power is 1.501 W. This solution is better suited for scenarios demanding strict calibration accuracy while accepting moderate resource overhead, e.g., industrial multi-sensor systems.

## 6. Conclusions

This paper presents a hybrid calibration method for nonlinear distortions and mismatch errors of TI-pipelined ADCs, ensembling a learned Volterra series with a neural network. By synergizing the interpretability of Volterra with the adaptive learning capability of neural networks, this method achieves high calibration performance while maintaining low computational complexity. This ensemble strategy not only improves cost efficiency but also offers a scalable solution for high-speed ADC calibration, advancing the integration of ML in mixed-signal design.

## Figures and Tables

**Table 1:** Calibration of Static Nonlinear Distortion of Sub-ADC with Respect to Order and Memory Depth.

Memory Depth	SFDR (First Order)	SFDR (Second Order)	SFDR (Third Order)	SFDR (Fourth Order)	SFDR (Fifth Order)
One	61.85 dB	64.37 dB	67.37 dB	70.38 dB	70.11 dB
Two	61.89 dB	65.91 dB	70.46 dB	70.83 dB	70.65 dB
Three	61.88 dB	64.78 dB	69.48 dB	70.39 dB	70.76 dB
Four	61.91 dB	65.43 dB	69.63 dB	69.99 dB	70.43 dB

**Table 2:** Comparison of calibration performance and model complexity of TIADCs.

	[7]	[17]	[19]	[15]	[31]	This Work	
ADC Architecture	Pipelined	TI-pipelined	TI-pipelined	TI-SAR	TI-SAR	TI-pipelined	TI-pipelined
Resolution (bit)	12	12	12	10	12	12	16
Sampling Rate (SPS)	800 M	600 M	5400 M	5000 M	3000 M	3000 M	1000 M
Channel	1	4	4	16	3	4	4
Calibration errors	Nonlinearity	Nonlinearity and Mismatch ^1^	Nonlinearity and Mismatch	Dynamic and Static Errors	Δo, Δg, Δt^2^	Nonlinearity and Mismatch	Nonlinearity and Mismatch
Normalized Frequency	N/A	N/A	[0 0.5]	N/A	N/A	[0 0.5]	[0 0.5]
Calibrator Type	DFT-LMS	FCNN-based	CNN-based	FCNN-based	REF-based	FCNN-based	FCNN-based
SFDR (dB)	72.1	71.23	78.25	72.50	70.0	79.70	80.90
SNDR (dB)	54.9	59.19	53.98	48.20	63.5	55.63	62.43
Parameters	-	13.19k	51.4k	24.61k	-	4.4k	4.4k
FLOPs	-	106.95M	103.55M	11.53M	-	8.57M	8.57M

^1^ Nonlinearity and Mismatch: Nonlinear distortions and Mismatch errors. ^2^ Δo, Δg, Δt: offset mismatch error, gain mismatch error, timing skew mismatch error.

**Table 3:** Comparison of calibration performance of Volterra model and ensemble model on TI-pipelined ADCs.

ADC Architecture	Method	SNDR (Before)	SNDR (After)	SFDR (Before)	SFDR (After)
TI-pipelined ADC1	V-model ^1^	35.35 dB	37.29 dB	35.47 dB	35.50 dB
TI-pipelined ADC1	E-model ^2^	37.29 dB	55.63 dB	35.50 dB	79.70 dB
TI-pipelined ADC2	V-model	40.21 dB	42.17 dB	38.62 dB	38.63 dB
TI-pipelined ADC2	E-model	42.17 dB	62.43 dB	38.63 dB	80.90 dB

^1^ V-model: Volterra model. ^2^ E-model: Ensemble model.

**Table 4:** FPGA resource utilization.

Resource	Utilization	Available	Utilization %
LUT	3480	341,280	1.02
LUTRAM	738	184,320	0.40
FF	28,800	682,560	4.22
DSP	328	3528	9.30

## Data Availability

The original contributions presented in this study are included in the article. Further inquiries can be directed to the author.
